# A novel double-lumen tracheal tube-assisted InSurE versus LISA in preterm infants with RDS: a multicenter randomized trial

**DOI:** 10.3389/fped.2026.1869213

**Published:** 2026-08-06

**Authors:** Jin Gao, Hanmei Xiong, Chuanfeng Li, Weihong Yu, Jie Yang, Panrong Nie, Lingyun Bao, Hong Yang, Yuan Shi

**Affiliations:** 1 Department of Neonatology, National Clinical Research Center for Children and Adolescents' Health and Diseases, Ministry of Education Key Laboratory of Child Development and Disorders, Children's Hospital of Chongqing Medical University, Chongqing, China; 2 Key Laboratory of Child Rare Diseases in Infection and Immunity, Children's Hospital of Chongqing Medical University, Chongqing, China; 3 Department of Neonatology, Kunming Children’s Hospital, Kunming, Yunnan, China; 4 Department of Neonatology, The First People’s Hospital of Zhaotong, Zhaotong, Yunnan, China; 5 Department of Neonatology, Qujing Maternity and Child Healthcare Hospital, Qujing, Yunnan, China; 6 Department of Neonatology, The People’s Hospital of Wenshan Prefecture, Wenshan, Yunnan, China; 7 Department of Neonatology, Yuxi Children’s Hospital, Yuxi, Yunnan, China; 8 Department of Neonatology, Baoshan People’s Hospital, Baoshan, Yunnan, China

**Keywords:** double-lumen tube, InSurE, LISA, neonatal respiratory distress syndrome, pulmonary surfactant

## Abstract

**Background:**

Neonatal respiratory distress syndrome (NRDS) is a leading cause of mortality and long-term morbidity in preterm infants; however, safe and effective surfactant delivery to the neonatal lung remains challenging. We developed a novel dual-lumen tracheal tube (NDT) enabling surfactant administration without interrupting ventilation. This study aimed to evaluate the non-inferiority of the novel NDT-based technique compared with the Less Invasive Surfactant Administration (LISA) technique in reducing the need for early invasive mechanical ventilation.

**Methods:**

The NDT, designed for a modified Intubation–Surfactant–Extubation (InSurE) technique (NDT-InSurE), features an embedded 0.2 mm channel. A multicenter, randomized controlled trial was conducted across six tertiary neonatal intensive care units (NICUs) in China. Preterm infants ≤ 32 weeks’ gestation with NRDS were randomly assigned to the NDT-InSurE group (*n* = 69) or the LISA group (*n* = 69). The primary outcome was the requirement for mechanical ventilation within 72 h of treatment initiation due to non-invasive ventilation failure. Secondary outcomes included key clinical outcomes and major complications associated with NRDS.

**Results:**

Baseline characteristics were comparable between the two groups. The primary outcome occurred in 10 infants (14.5%) in the NDT-InSurE group and 13 (18.8%) in the LISA group (*P* = 0.493; 95% CI, −12.5% to 4.0%). The upper bound of the 95% confidence interval was below the prespecified non-inferiority margin of 20%, indicating that the NDT-InSurE technique met the prespecified criterion for non-inferiority. Secondary outcomes, including total procedure duration, intubation time, duration of invasive ventilation, non-invasive ventilation, oxygen therapy, and total hospitalization time, showed no significant differences between the two groups (all *P* > 0.05). However, post-intubation arterial pH was lower in the NDT-InSurE group than in the LISA group (7.27 [7.25–7.35] vs. 7.38 [7.32–7.42], *P* = 0.004). Moreover, the incidence of major complications, including bronchopulmonary dysplasia, intraventricular hemorrhage, retinopathy of prematurity, and necrotizing enterocolitis, did not differ significantly between groups.

**Conclusions:**

NDT-InSurE may serve as a practical alternative to LISA, particularly in settings where LISA expertise or resources are limited. Further large-scale, multicenter studies with long-term follow-up are warranted to confirm these findings and further evaluate the efficacy and safety of this technique.

**Clinical Trial Registration:**

This study was registered at the China Clinical Trial Registry (https://www.chictr.org.cn/), Registration Number: ChiCTR2300076354.The registration date is October 7th, 2023.

## 1 Introduction

Neonatal respiratory distress syndrome (NRDS) is a major cause of respiratory morbidity in preterm infants (1). Non-invasive ventilation combined with pulmonary surfactant (PS) therapy is recommended by current guidelines and widely used in clinical practice for NRDS (2–4). Among PS delivery methods, the Intubation-Surfactant-Extubation (InSurE) and Less Invasive Surfactant Administration (LISA) techniques have garnered significant attention (5–7). LISA employs a specialized thin catheter to administer surfactant, allowing patients to maintain spontaneous breathing under continuous positive airway pressure (CPAP). This approach has been shown to improve long-term respiratory outcomes and reduce the incidence of bronchopulmonary dysplasia (BPD) (8). Moreover, Budajaja et al. (9) reviewed multiple clinical studies and meta-analyses, demonstrating that LISA is associated with a reduced need for invasive ventilation and a lower risk of BPD compared with the conventional InSurE technique. Despite these advantages, LISA has not yet been widely adopted worldwide. This may be attributed to several practical challenges, including procedural complexity, the occasional need for Magill forceps to facilitate catheter placement, and the relatively high cost of specialized thin catheters in certain regions, which may limit its use in resource-limited settings (8, 10). Consequently, the InSurE technique remains commonly used in many neonatal intensive care units (NICUs) due to its relative simplicity and lower equipment requirements, making it a practical alternative when LISA is not feasible.

However, the InSurE technique is not without limitations, as surfactant administration and positive pressure ventilation share the same lumen, which can transiently impede airflow and compromise gas exchange during surfactant delivery. To address this issue, we developed a novel dual-lumen tracheal tube (NDT) that enables simultaneous ventilation and surfactant administration by separating the pathways for airflow and drug delivery. We therefore conducted a multicenter, randomized controlled trial in preterm infants with NRDS (≤ 32 weeks' gestation) to evaluate whether the NDT-based InSurE technique is non-inferior to LISA. This study provides preliminary evidence regarding the potential role of the NDT-based technique in settings where LISA implementation may be limited by technical complexity or resource constraints.

## 2 Materials and methods

### 2.1 Design, structure, and clinical application of the NDT

The NDT used in this study was designed by our research team (Patent No. CN 209645598 U, November 2019) and manufactured by Tuoren Medical (Registration No. 20172660345; Henan, China), based on the clinical requirement for simultaneous drug delivery and ventilation in the treatment of NRDS, representing a device-based innovation for simultaneous ventilation and drug delivery.

**Figure 1:**
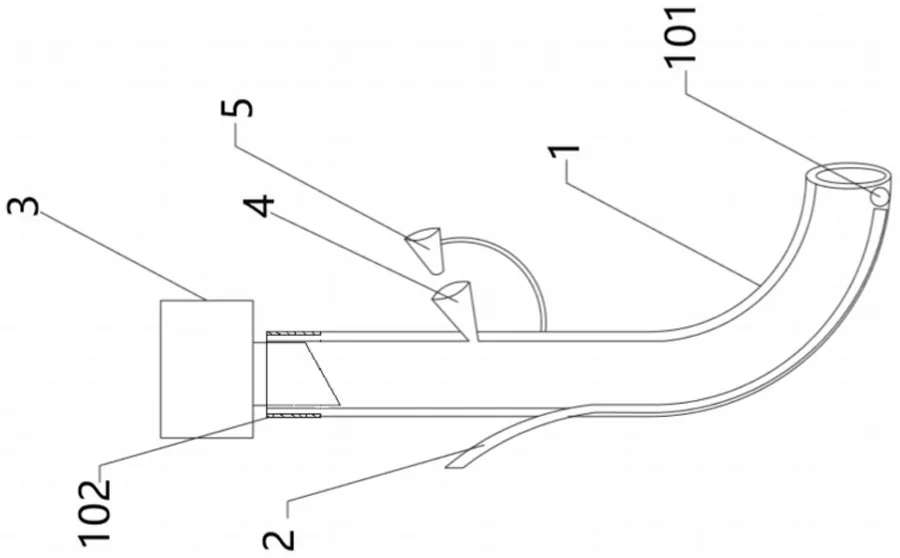
Initial design of the novel dual-lumen tracheal tube. (1) Main tracheal tube, with total length designed according to neonatal airway dimensions. (2) Drug delivery channel, a microchannel with an inner diameter of approximately 0.2 mm, running longitudinally along the inner wall of the main tube. (3) Connector. (4, 5) Suction catheter port and sealing plug. (101) Murphy eye, where the distal opening of the drug delivery channel is located. (102) Anti-slip grooves.

In clinical application, the NDT is inserted into the trachea via the oral or nasal route and connected through the connector (3) to a ventilator or T-piece resuscitator to provide CPAP. For surfactant administration, a syringe prefilled with PS is attached to the proximal end of the drug delivery channel (2), and PS is slowly injected. The surfactant is delivered through the channel to the Murphy eye (101) and subsequently transported by ventilatory airflow into the distal airways (Figure 1).

**Figure 2:**
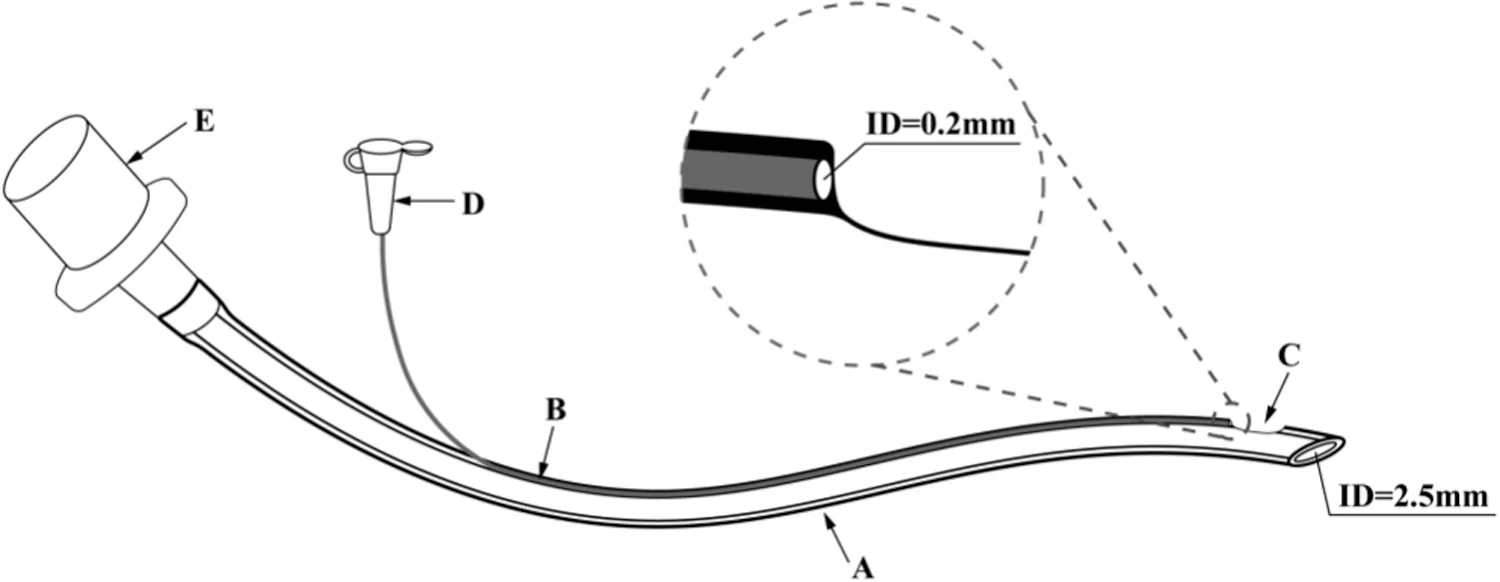
The new double-lumen tracheal tube diagram (take model 1 as an example). ID: Inner diameter. **(A)**The dominant tube, whose diameter is chosen according to the weight of the newborn (Model 1: ID 2.5mm: Model 2: ID 3.0 mm; Model 3: ID 3.5mm: Model 4: ID 4.0 mm). **(B)** The drug delivery tube located inside the wall of the dominant tube, with an ID of 0.2 mm. **(C)**The Murphy Eye in direct communication with the distal end of the drug delivery tube. **(D)**The plastic cap for closing the drug injection port. **(E)** The tube connector, Reprinted from Li et al. (2022), Frontiers in Pediatrics, DOI: doi: 10.3389/fped.2022.1032044. Licensed under CC BY 4.0. With permission from the corresponding author.

The initial design is shown in Figure 1, and the final optimized product is presented in Figure 2 (11) following minor iterative refinements with the manufacturer. This configuration separates the pathways for ventilation and surfactant delivery, allowing continuous positive pressure ventilation during drug administration.

### 2.2 Study design and participants

This study was a researcher-initiated, multicenter, randomized controlled trial conducted in six tertiary NICUs in Yunnan Province, China (Kunming, Wenshan, Qujing, Zhaotong, Baoshan, and Yuxi), between January 1, 2024, and January 31, 2025. Participants were randomly assigned to receive pulmonary surfactant (PS) via either NDT-InSurE or LISA. The study protocol was registered at the Chinese Clinical Trial Registry (https://www.chictr.org.cn/) with registration number ChiCTR2300076354. The trial protocol, including the assessment of outcomes at 1-year corrected age, was approved by the ethics committees of all participating centers. Kunming Children's Hospital, as the initiating institution, obtained approval from its Medical Ethics Committee (approval number: 2023-03-297-K01). Prospective written informed consent was obtained from parents for all trial procedures.

### 2.3 Eligibility criteria

Neonates with a gestational age ≤ 32 weeks and admitted to any participating NICUs within 6 h of birth were eligible for inclusion. Inclusion criteria were: (1) Requirement for non-invasive respiratory support with a fraction of inspired oxygen (FiO₂) ≥ 0.30 to maintain saturation of peripheral oxygen (SpO₂) between 90% and 95%; (2) Clinical and radiographic evidence of NRDS; (3) Written parental consent obtained.

Exclusion criteria included: life-threatening congenital anomalies; complex cyanotic congenital heart disease; pulmonary hypoplasia; chromosomal abnormalities; congenital metabolic defects; neuromuscular disorders affecting respiration; grade III or higher intraventricular hemorrhage on early cranial ultrasound; requirement for endotracheal intubation and mechanical ventilation immediately after birth or due to urgent clinical need; requirement for surgery within 72 h; or transfer to another facility prior to randomization (12).

### 2.4 Intervention measures

The design of the intervention measures, as well as the procedures for randomization and blinding, has been described previously (13). When an FiO₂ of ≥0.30 was required to maintain the target SpO₂, neonates were assigned to receive surfactant therapy using either NDT-InSurE or LISA. The LISA group used a standardized LISA catheter (Jiulong Medical Devices Co., Ltd., Wuxi, China). Infants in both groups used PS according to the method described in the previously published protocol (4). In the NDT-InSurE group, positive pressure ventilation was provided through T-piece.No sedation, analgesia, muscle relaxants, or premedication were administered before or during the procedures in either group, in accordance with the predefined study protocol across all participating centers.

Both groups received surfactant via endotracheal administration: porcine pulmonary surfactant (Casi Pharmaceuticals, Italy) at 200 mg/kg, or bovine pulmonary surfactant (China Resources Double Crane Pharmaceutical Co., Ltd., Beijing, China) at 100 mg/kg. A second dose was administered if clinically indicated, with a maximum of four doses using the same method as the initial dose. Following surfactant intervention, non-invasive respiratory support was continued unless intubation criteria were met.

Non-invasive ventilation failure was defined as any of the following within 72 h after birth, prompting immediate mechanical ventilation (13, 14): (1) Severe respiratory acidosis (PaCO₂ > 60 mmHg, pH < 7.2) confirmed by two arterial blood gas samples obtained more than 30 min apart; (2) Frequent apnea (>3 episodes/h) unresponsive to medication or nCPAP; (3) Persistent hypoxemia (PaO₂ < 50 mmHg or SpO₂ < 85% despite FiO₂ ≥ 0.5); (4) Cardiopulmonary arrest requiring endotracheal intubation for resuscitation, or any type of neonatal pulmonary hemorrhage; (5) Other clinical emergencies.

### 2.5 Primary and secondary outcomes

Primary outcome: Need for mechanical ventilation within 72 h after the start of treatment, due to failure of non-invasive ventilation.

Secondary outcomes: (1) Duration of mechanical ventilation, non-invasive ventilation, oxygen therapy, and hospital stay; (2) Total procedure time (from intubation initiation to catheter removal after surfactant infusion), number of intubation attempts before successful surfactant delivery, and incidence of uneven surfactant distribution (4). (3) Incidence of adverse events during administration, including bradycardia (heart rate < 100 beats/min), tachycardia (heart rate > 200 beats/min), hypoxemia (SpO₂ ≤ 80%), and oral mucosal or gingival injury; (4) Pre- and post-surfactant values of SpO₂, FiO₂, pH, PaCO₂, and PaO₂; (5) Major complications, including pneumothorax, neonatal necrotizing enterocolitis (NEC) (Bell stage 2 or higher) (15, 16), retinopathy of prematurity (ROP) (defined as grades 2–4 based on the International Classification) (17), intraventricular hemorrhage (IVH) (Papile classification grades 3–4) (18), periventricular leukomalacia (PVL), pulmonary hemorrhage, BPD at 36 weeks' corrected gestational age (2001 NICHD criteria) (19, 20), and mortality.

### 2.6 Data collection

**Figure 3:**
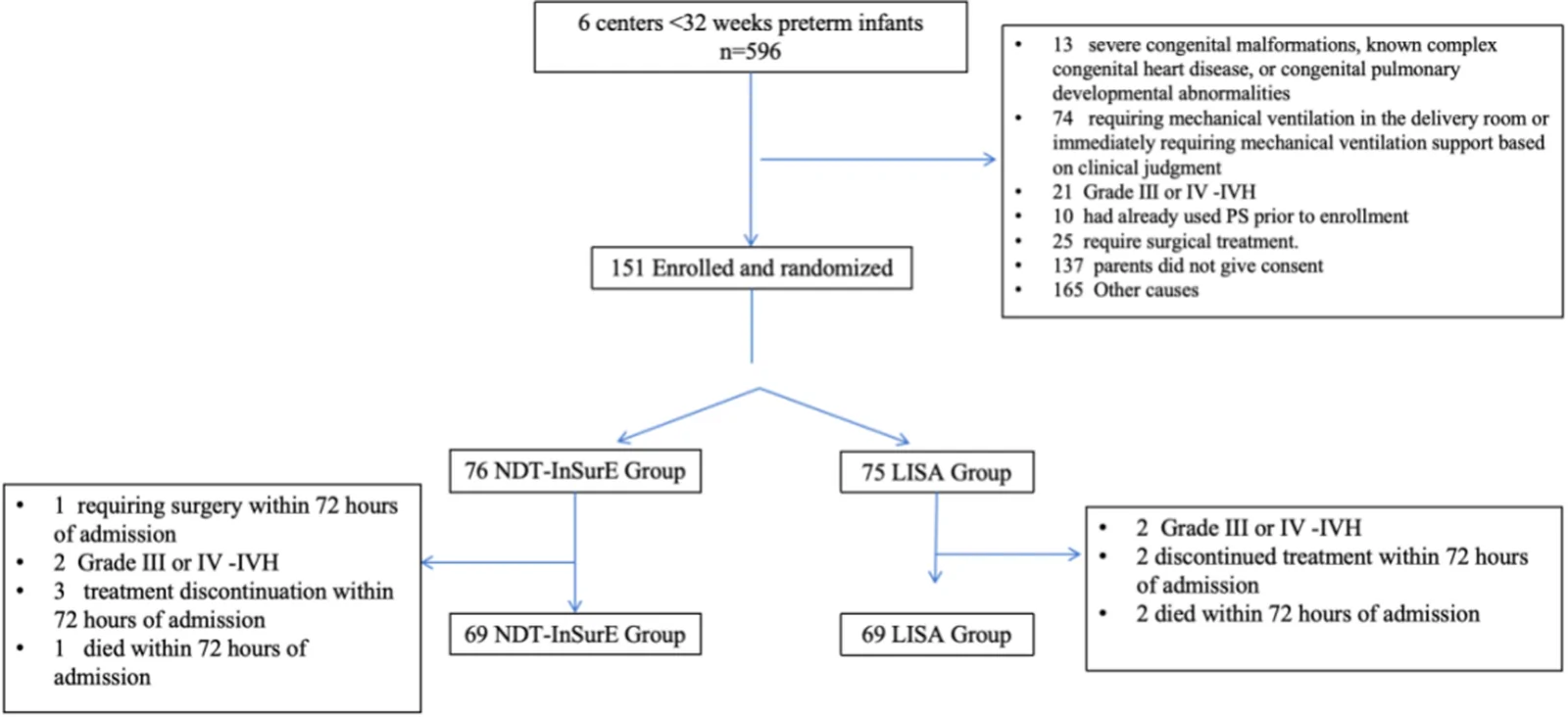
Flow diagram of screening. enrollment, and randomization.

All outcome data were collected by personnel blinded to group allocation, and parents were also blinded to the assigned intervention. Data were initially obtained and recorded by attending physicians, then verified and entered into statistical tables by team members not involved in patient care. Preliminary statistical analysis was conducted when enrollment exceeded 50% of the planned sample. No significant differences were observed between groups, allowing the study to proceed until data collection completion (Figure 3).

### 2.7 Sample size

The sample size was estimated based on the primary outcome: requirement for mechanical ventilation within 72 hours after the start of treatment due to failure of non-invasive ventilation. According to published data, the incidence of this outcome is approximately 30% among preterm infants with gestational age < 32 weeks who receive non-invasive respiratory support (21, 22). A non-inferiority margin of 20% for the absolute risk difference was prespecified. Sample size calculation was performed using PASS software (V.15.0) for a one-sided test with *α* = 0.025 and powe*r* = 80%, assuming an event rate of 30% in both groups.The calculated sample size was 59 infants per group. After accounting for potential missed diagnoses, incomplete data, and early withdrawal, the target sample size was increased to at least 66 infants per group. Ultimately, 69 infants were enrolled in each group, resulting in a total sample size of 138 infants. The resulting statistical power was approximately 75%, which remains sufficient to test the pre-defined non-inferiority hypothesis.

### 2.8 Statistical analysis

Data analysis was performed using SPSS software (V. 27.0). Normally distributed variables were presented as mea*n* ± standard deviation (SD), and pairwise comparisons were performed using Student's t-test. Non-normally distributed variables were presented as median (interquartile range, IQR), and were compared using the Mann–Whitney *U*-test. Categorical variables were expressed as counts (percentages) and were compared using chi-square (*χ*^2^) test.

Non-inferiority of the NDT-InSurE technique compared with LISA was assessed using the primary outcome. Two-sided 95% confidence intervals (CIs) for the risk difference were calculated, and non-inferiority was established if the lower bound exceeded the pre-specified margin of −20%. Secondary outcomes were analyzed using the conventional statistical methods described above, and *P* < 0.05 was considered statistically significant.

## 3 Results

### 3.1 Baseline characteristics

Among the 596 newborns screened at the six participating NICU centers, 151 were randomly assigned, with 138 ultimately completing the study. Of the 138 enrolled infants, 69 were assigned to the NDT-InSurE group and 69 to the LISA group (Figure 2). No significant differences were observed between groups in birth weight, gestational age, Apgar scores at 1 and 5 min, *in vitro* fertilization (IVF) status, method of delivery, and antenatal glucocorticoids use. Moreover, no significant differences were observed regarding the type of PS administered, time to first PS administration, and time to initiation of non-invasive ventilation after birth (all *P* > 0.05). The only significant difference was maternal age, which was slightly higher in the LISA group compared with the NDT-InSurE group (30.09 ± 4.89 years vs. 27.50 ± 5.86 years; *P* = 0.006 (Table 1).

**Table 1:** Baseline characteristics of enrolled neonates in the NDT-inSurE and LISA groups.

Characteristics		NDT-InSurE group (*n* = 69)	LISA group (*n* = 69)	Test statistic	*P*-value
Birth weight, g		1,381.14 ± 262.28	1,343.49 ± 300.58	t = 0.785	0.434
Gestational age, week, median（P25, P75）		30.1 (29, 30.3)	30.2 (28.8, 31.2)	Z = −0.486	0.627
1-min Apgar, median (P25, P75)		8 (6, 8)	8 (6, 7)	Z = −0.201	0.841
5-min Apgar, median (P25, P75)		8 (7, 9)	9 (8, 10)	Z = −1.216	0.224
Maternal age, years		27.50 ± 5.860	30.09 ± 4.89	t = −2.806	0.006
Gender, *n* (%)	Male	32 (46.4)	26 (37.7)	*χ*^2^ = 1.071	0.301
	Female	37 (53.6)	43 (62.3)		
IVF, *n* (%)	Yes	29 (42)	22 (31.9)	χ^2^ = 1.524	0.571
	No	40 (58)	47 (68.1)		
Method of delivery *n* (%)	Vaginal delivery	32 (46.4)	38 (55.1)	χ^2^ = 1.549	0.667
	Cesarean section	37 (53.6)	31 (44.9)		
Single pregnancy *n* (%)	Yes	54 (78.3)	57 (82.6)	χ^2^ = 0.431	0.520
	No	15 (21.7)	12 (17.4)		
Birth order, *n* (%)	First child	17 (24.6)	13 (18.8)	χ^2^ = 0.681	0.409
	Not the first child	52 (75.4)	56 (81.2)		
Antenatal glucocorticoids, *n* (%)	Yes	45 (65.2)	51 (73.9)	χ^2^ = 1.232	0.267
	No	24 (34.8)	18 (26.1)		
Premature rupture of membranes, *n* (%)	Yes	46 (66.7)	40 (58)	χ^2^ = 1.111	0.292
	No	23 (33.3)	29 (42)		
Maternal educational background, *n* (%)	≥Junior High School	60 (87)	64 (92.8)	χ^2^ = 1.272	0.259
	< Junior High School	9 (13)	5 (7.2)		
Gestational diabetes, *n* (%)	Yes	24 (34.8)	32 (46.4)	χ^2^ = 1.923	0.165
	No	45 (65.2)	37 (53.6)		
Gestational hypertension, *n* (%)	Yes	32 (46.4)	27 (39.1)	χ^2^ = 0.740	0.390
	No	37 (53.6)	42 (60.9)		
Types of PS, *n* (%)	Calsurf	61 (88.4)	64 (92.8)	χ^2^ = 0.764	0.382
	Curosurf	8 (11.6)	5 (7.2)		
Age at first non-invasive ventilation, min, median (P25, P75)		23 (15, 41)	23 (14, 39)	Z = −0.230	0.818
Age at first PS administration, min, median (P25, P75)		120 (88.5, 190)	129 (81, 213)	Z = −0.006	0.995

Continuous variables with normal distribution are presented as mean ± SD and compared using Student's *t*-test. Continuous variables with non-normal distribution are presented as median (P25, P75) and compared using the Mann–Whitney *U*-test. Categorical variables are presented as *n* (%) and compared using the χ^2^ test. A *P*-value < 0.05 was considered statistically significant. IVF, *in vitro* fertilization; PS, pulmonary surfactant.

### 3.2 Primary outcome

Among enrolled neonates, 10 patients (14.5%) in the NDT-InSurE group and 13 (18.8%) in the LISA group required mechanical ventilation due to failure of non-invasive ventilation within 72 h, with a risk difference of −4.3% (95% CI, −12.5% to 4.0%; *P* = 0.493). The upper bound of the 95% CI (4.0%) was lower than the non-inferiority margin of 20%, indicating that NDT-InSurE met the criterion for non-inferiority compared with LISA (Table 2).

**Table 2:** Primary outcome in the NDT-inSurE and LISA groups.

Group (*n* = 138)	Invasive ventilation *n* (%)	Non-invasive ventilation *n* (%)	Test statistic	*P*-value	95% CI
NDT-InSurE (*n* = 69)	10 (14.5)	59 (85.5)	χ^2^ = 0.47	0.493	−4.3% (−12.5% to 4.0%)
LISA (*n* = 69)	13 (18.8)	56 (81.2)			

Considering that the effectiveness of surfactant delivery strategies may vary according to gestational age, we conducted subgroup analysis stratified by gestational age. Infants were classified into three gestational age groups: ≤28 weeks, >28–30 weeks, and >30–32 weeks. No significant between-group differences were observed in the primary outcome of mechanical ventilation due to non-invasive ventilation failure within 72 h in any gestational age subgroup (Table 3).

**Table 3:** Primary outcomes in NDT-inSurE and LISA groups across gestational age subgroups.

GA	Characteristics	NDT-InSurE group (*n* = 69)	LISA group (*n* = 69)	X^2^/fisher	*P*-value
≤28w	Invasive ventilation n(%)	1 (20)	4 (40)	Fisher	0.600
	Non-invasive ventilation n(%)	4 (80)	6 (60)		
>28–30 weeks	Invasive ventilation n(%)	6 (22.2)	5 (21.7)	0.002	0.967
	Non-invasive ventilation n(%)	21 (77.8)	18 (78.3)		
>30–32 weeks	Invasive ventilation n(%)	3 (8.1)	4 (11.1)	0.001	0.970
	Non-invasive ventilation n(%)	34 (91.9)	32(88.9)		

### 3.3 Secondary outcomes

No statistically significant differences were observed between the two groups in the duration of invasive ventilation, non-invasive ventilation, oxygen inhalation, or in the total length of hospital stay. The total procedure duration (from the start of intubation to catheter removal after surfactant administration) did not differ between groups, with 18 (10, 25) min in the NDT-InSurE group and 20 (10, 30) min in the LISA group (*P* = 0.375). Intubation time was also comparable between groups: 10 (6, 15) s vs. 10 (8, 20) s (*P* = 0.177).

Oxygenation and ventilation-related outcomes, including surfactant distribution, SpO₂ before and after PS administration, FiO₂, and blood gas results, were similar between the two groups. No significant differences were observed in adverse events occurring during intubation, such as bradycardia (heart rate <100 beats/min), tachycardia (heart rate >200 beats/min), hypoxemia (SpO₂ ≤ 80%), and oral mucosa or gingival injury.

Regarding complications, the incidence of BPD was slightly higher in the NDT-InSurE group than in the LISA group, but the difference was not statistically significant (30.4% vs. 23.2%, *P* = 0.442). Other complications, including ROP, IVH, pneumothorax, NEC, PVL, and pulmonary hemorrhage, occurred in both groups, but with no significant differences (all *P* > 0.05) (Table 4).

**Table 4:** Secondary outcomes in the NDT-inSurE and LISA groups.

Parameters	NDT-InSurE group (*n* = 69)	LISA group (*n* = 69)	Test statistic	*P*-value
Procedure duration				
Total procedure duration, min, median (P25, P75)	18 (10, 25)	20 (10, 30)	Z = −0.887	0.375
Intubation time, s, median (P25, P75)	10 (6, 15)	10 (8, 20)	Z = −1.350	0.177
Invasive ventilation duration, h	210.9 ± 193.2	165.9 ± 90.9	t = 1.500	0.493
Non-invasive ventilation duration, h	364.2 ± 338.6	404.6 ± 397.1	t = −0.561	0.582
Oxygen therapy duration, h	167.0 ± 332.9	418.9 ± 307.9	t = −2.694	0.066
Total hospitalization time, d	53.2 ± 23.3	51.7 ± 26.7	t = 0.320	0.703
Ventilation and oxygenation				
SpO_2_ after PS, %	93.58 ± 1.59	93.48 ± 1.41	t = 0.306	0.761
FiO2 before PS, %	42.77 ± 20.54	38.43 ± 16.04	t = 1.343	0.182
FiO2 after PS, %	38.96 ± 21.92	35.29 ± 16.78	t = 1.024	0.308
pH before PS, median (P25, P75)	7.29 (7.22, 7.32)	7.34 (7.3, 7.4)	t = −1.434	0.152
pH after PS, median (P25, P75)	7.27 (7.25, 7.35)	7.38 (7.32, 7.42)	t = −2.860	0.004
PaO₂ before PS, mmHg, median (P25, P75)	88.9 (74, 111)	8 9 (59, 106)	Z = −0.808	0.423
PaO₂ after PS, mmHg, median (P25, P75)	75 (59.8, 85.9)	85 (59, 99)	Z = −1.100	0.271
PaCO₂ before PS, mmHg, median (P25, P75)	38.3 (31.9, 52)	38.1 (34, 47.2)	Z = −0.504	0.614
PaCO₂ after PS, mmHg, median (P25, P75)	34 (28.3, 39)	32 (25, 36.2)	Z = −1.213	0.225
Adverse events, *n* (%)				
Bradycardia	6 (8.7)	1 (1.4)	χ^2^ = 3.672	0.115
Tachycardia	0	2 (2.9)	Fisher Fisher's exact test	0.496
Hypoxemia	3 (4.3)	2 (2.9)	Fisher Fisher's exact test	1.000
Oral mucosal injury	0	0	-	-
Uneven PS administration	8 (11.6)	11 (15.9)	χ^2^ = 0.549	0.622
Complications, *n* (%)				
Pneumothorax	7 (10.1)	10 (14.5)	χ^2^ = 0.604	0.606
NEC	7 (10.1)	4 (5.8)	χ^2^ = 0.889	0.532
ROP	22 (31.9)	26 (37.7)	χ^2^ = 0.511	0.592
IVH	14 (20.3)	12 (17.4)	χ^2^ = 0.190	0.828
PVL	10 (14.5)	7 (10.1)	χ^2^ = 0.604	0.606
Pulmonary hemorrhage	5 (7.2)	7 (10.1)	χ^2^ = 0.365	0.764
BPD	21 (30.4)	16 (23.2)	χ^2^ = 0.932	0.442

Data are presented as mean ± SD, median (P25, P75), or *n* (%). Independent samples *t*-test, Mann–Whitney *U*-test, Chi-square test, or Fisher's exact test was used where appropriate. *P* < 0.05 was considered statistically significant.

BPD, bronchopulmonary dysplasia; FiO2, fraction of inspired oxygen; IVH, intraventricular hemorrhage; NEC, necrotizing enterocolitis; PaCO2, partial pressure of carbon dioxide; PaO2, partial pressure of oxygen; PS, pulmonary surfactant; PVL, periventricular leukomalacia; ROP, retinopathy of prematurity; SpO2, peripheral oxygen saturation.

Among subgroups stratified by gestational age, no statistically significant differences were observed between the two groups in the ≤28-week subgroup in duration-related indicators, including total procedural duration, intubation time, invasive ventilation duration, non-invasive ventilation duration, oxygen supplementation duration, and total hospital stay. A statistically significant difference in post-procedure FiO₂ was observed between the two groups (*P* = 0.038) (Supplementary Materials Table S1).

In the >28–30-week subgroup, the duration of invasive ventilation was significantly longer in the LISA group [375 (287, 406) h] than in the NDT-InSurE group [233 (214, 323) h] (*P* < 0.001). In addition, post-surfactant arterial blood gas pH was lower in the NDT-InSurE group [7.3 (7.26, 7.36)] compared with the LISA group [7.36 (7.32, 7.41)] (*P* = 0.005) (Supplementary Materials Table S2).

In the >30–32-week subgroup, the total oxygen therapy duration was slightly shorter in the NDT-InSurE group [582 (128, 654) h] relative to the LISA group [683 (176, 758) h], with a statistically significant difference (*P* = 0.002). A similar difference in post-surfactant pH was observed between groups (*P* = 0.007) (Supplementary Materials Table S3).

## 4 Discussion

In this multicenter randomized trial of preterm infants born at ≤32 weeks’ gestation with NRDS, NDT-InSurE was non-inferior to LISA with respect to the incidence of invasive mechanical ventilation within 72 h due to non-invasive ventilation failure. Our study provides preliminary evidence supporting the use of NDT in the InSurE technique. Similar patterns were observed across gestational age subgroups. These findings suggest that NDT-InSurE may offer a practical alternative in clinical settings where LISA expertise is limited or where resource and economic constraints limit its routine implementation, rather than a replacement for standard LISA in centers where the technique is well established.

The combination of non-invasive ventilation and surfactant administration is widely accepted as a standard approach for the management of NRDS in preterm infants (23). PS can be administered via multiple routes, and current guidelines recommend device-delivered PS based on provider experience, preference, and institutional practice (24). Historically, the InSurE technique was widely used in clinical practice (25). In recent years, minimally invasive administration approaches represented by LISA have gradually become a hotspot in clinical research and application due to their advantages. The relevant European guidelines in 2022 (23) explicitly recommended LISA as the preferred method of PS administration for spontaneously breathing preterm infants supported by CPAP, as it reduces the rate of invasive ventilation and related complications. The clinical efficacy of LISA has been verified in multiple studies. A meta-analysis by Huo et al. (26), including nine randomized controlled trials with a total of 1,212 infants, showed that the rate of mechanical ventilation within 72 h was significantly lower in the LISA group (OR = 0.39, 95% CI: 0.29–0.51, *P* < 0.001). Anand et al. (27) also reported a lower rate of CPAP failure in the LISA group compared with InSurE group (RR = 0.55, 95% CI: 0.34–0.89, *P* = 0.005). Evidence suggests that the efficacy of LISA in preventing mechanical ventilation within 72 h after birth is closely related to gestational age, with preterm infants of 26–30 benefiting the most (28). We stratified 138 enrolled infants into three subgroups based on gestational age: ≤28 weeks, >28–30 weeks, and >30–32 weeks. No statistically significant differences were observed in the primary outcome across gestational age subgroups. This study demonstrates that NDT-InSurE achieved similar short-term outcomes compared with LISA. This may be attributable to the innovative design of the NDT (12), which enables PS delivery directly to the distal opening through an embedded fine lumen under continuous ventilation. By separating the PS delivery pathway from the positive pressure ventilation pathway, the NDT may overcome key limitations of the traditional InSurE technique, consistent with the core concept of “uninterrupted” treatment of LISA.

In this study, the median time from birth to initiation of non-invasive ventilation was 23 (15, 41) min in the NDT-InSurE group and 23 (14, 39) min in the LISA group. The median time to first surfactant administration was 120 (88.5, 190) min and 129 (81, 213) min, respectively. No significant differences were observed for either parameter. This suggests that any potential differences in outcomes were mainly driven by differences in PS administration techniques rather than disparities in the timing of initial treatment. A previous multicenter study by Liu et al. (29) reported significantly longer drug instillation and total procedure times in the minimally invasive surfactant administration group compared with the conventional tracheal intubation group [60 (18, 270) s vs. 50 (30, 60) s; 90 (60, 300) s vs. 60 (44, 270) s; Z = 3.009, 3.365; *P* = 0.003, 0.001]. In contrast, our study showed no significant differences in catheter insertion or total procedure duration between the NDT-InSurE group and the LISA group [10 (6, 15) s vs. 10 (8, 20) s; 18 (10, 25) min vs. 20 (10, 30) min; Z = 1.350, 0.887; *P* = 0.177, 0.375]. These findings may be partly explained by operator experience. In the NDT-InSurE group, operators comprised 21 junior, 34 intermediate, and 14 senior professionals, compared with 18, 41, and 10 in the LISA group, respectively. The proportion of intermediate and senior operators was 69.6% and 73.9%, respectively, and their extensive experience may have facilitated smooth catheter insertion. In addition, the total procedure duration in our study (18–20 min) was longer than that reported by Liu et al. (60–90 s), which is associated with the clinical management strategies adopted by the participating institutions in this study. In the participating centers, surfactant was administered more slowly to reduce reflux. Only 12 of the 138 infants experienced PS reflux, which may support the feasibility of this strategy. To our knowledge, no studies have compared the clinical effects of different PS delivery rates, and the present study may provide a reference for future research. A study by Mishra et al. (21) showed no significant differences in mean duration of oxygen therapy (172.3 vs. 179.7 h, *P* = 0.13) or length of hospital stay (20.5 vs. 22.2 days, *P* = 0.12) between the thin catheter group and the conventional InSurE group. Similarly, the present study found no statistically significant differences in total duration of invasive ventilation, duration of non-invasive ventilation, duration of oxygen therapy, or length of hospital stay between the NDT-InSurR and LISA groups. These findings further suggest that the NDT-InSurE technique was associated with short-term clinical outcomes consistent with those of LISA within the prespecified non-inferiority framework, supporting its potential use in selected clinical settings.

Among subgroups stratified by gestational age, infants born at ≤28 weeks exhibited lower post-surfactant FiO₂ in the LISA group (Supplementary Materials Table S5). This observation may be related to the use of a thin catheter in the LISA procedure, which allows minimal airway irritation while maintaining spontaneous breathing, thereby reducing the risk of lung injury. This finding may suggest a potential procedural advantage of LISA in extremely preterm infants, although this requires further confirmation. No significant differences were observed in other secondary outcomes related to procedural duration, ventilation and oxygenation parameters.

In our study, analysis of blood oxygen and blood gas parameters before and after administration in the two groups showed no significant differences in SpO₂, FiO₂, PaO₂, and PaCO₂. Although the post-treatment pH in the NDT-InSurE group was slightly lower than that in the LISA group [7.27 (7.25, 7.35) vs. 7.38 (7.32, 7.42), Z = −2.86, *P* = 0.004], it remained within the normal physiological range for preterm infants within the first 24 h after birth. Clinically, these findings indicate no meaningful differences between the two groups. Consistent findings were observed in the >28–30-week and >30–32-week gestational age subgroups (Supplementary Materials Table S1-S3). This is consistent with the study by Han et al. (30), which reported no significant differences in blood gas parameters—including pH, PaO₂, PaCO₂, and SaO₂—at baseline, 1 h, and 12 h after surfactant administration between the thin catheter group and the conventional InSurE group.

No significant differences were observed between the two groups in the incidence of adverse reactions during catheter insertion, including oral mucosal injury, bradycardia, tachycardia, and hypoxemia, consistent with previous reports (21, 31). Similarly, the incidence of uneven PS administration did not differ between groups. These findings suggest that the novel tube, which delivers surfactant through a channel embedded in the catheter wall, may facilitate effective surfactant delivery and was associated with short-term clinical outcomes consistent with those achieved using a LISA catheter.

Several studies have evaluated minimally invasive thin-catheter surfactant administration compared with the conventional InSurE technique. The OPTIMIST-A trial by Dargaville et al. (32) showed that compared with the control group, minimally invasive surfactant administration did not significantly reduce the composite outcome of death or bronchopulmonary dysplasia at a postmenstrual age of 36 weeks, but it significantly reduced the incidence of BPD at 36 weeks postmenstrual age. A 2021 Cochrane meta-analysis (31), including 16 trials involving 2,164 neonates, showed that the thin catheter group significantly reduced the risk of multiple key adverse outcomes: a 41% reduction in the composite risk of death or BPD at 36 weeks postmenstrual age (RR 0.59, 95% CI 0.48–0.73); a 37% reduction in the risk of intubation within 72 h after birth (RR 0.63, 95% CI 0.54–0.74); a 37% reduction in the risk of severe intraventricular hemorrhage (grade III/IV) (RR 0.63, 95% CI 0.42–0.96); a 37% reduction in the risk of in-hospital mortality (RR 0.63, 95% CI 0.47–0.84); and a 43% reduction in the risk of BPD among survivors (RR 0.57, 95% CI 0.45–0.74). In a 2025 study by Cho et al. (33), which enrolled 1,112 preterm infants (gestational age <32 weeks) in South Korea, the LISA group demonstrated significantly lower risks of massive pulmonary hemorrhage (aOR 0.314), patent ductus arteriosus (PDA) (aOR 0.67), sepsis (aOR 0.607), severe BPD (aOR 0.514), and the composite outcome of severe BPD or death (aOR 0.586), as compared with the InSurE group. In the present study, no statistically significant differences were observed between NDT-InSurE and LISA regarding major neonatal complications, including pneumothorax, severe IVH (grade 3–4), pulmonary hemorrhage, NEC, ROP, and BPD. Although the sample size limits the statistical power to detect differences in these low-incidence outcomes, no evidence of between-group differences was observed across these outcomes. However, these findings should be interpreted as exploratory and require confirmation in larger studies.

Despite the strengths of this study, including its randomized design and multicenter participation, several limitations should be acknowledged. First, the prespecified non-inferiority margin of 20% is relatively wide in relation to the observed event rate, which may limit the clinical interpretability of the findings. Future adequately powered trials should adopt a more stringent non-inferiority margin. Second, the observed incidence of the primary outcome was lower than expected, possibly because the enrolled infants had a relatively mature gestational age, further reducing the effective statistical power. Consequently, the study may have been underpowered to detect differences in relatively infrequent secondary outcomes, including bronchopulmonary dysplasia, intraventricular hemorrhage, necrotizing enterocolitis, and mortality. Therefore, these secondary outcomes should be considered exploratory and interpreted with caution. Third, formal assessments of procedural pain, stress, and patient tolerance using validated scoring systems were not performed. Although no obvious differences were observed between groups in procedural duration or procedure-related adverse events, these measures are indirect and cannot fully reflect procedural tolerance. Finally, neurodevelopmental and motor outcomes were not evaluated, limiting assessment of the long-term safety and efficacy of the NDT-InSurE technique. Nevertheless, PS administration via the NDT demonstrates a safe and effective profile, with non-inferior efficacy compared with LISA. These findings provide preliminary support for its clinical application, and larger, multicenter randomized trials with extended follow-up are warranted to strengthen evidence on complications, pulmonary function, neurodevelopment, and growth in preterm infants, thereby supporting broader implementation of the NDT-InSurE technique.

## 5 Conclusions

In preterm infants with a gestational age ≤ 32 weeks diagnosed with NRDS, the NDT-InSurE technique was non-inferior to LISA in terms of the incidence of invasive mechanical ventilation due to non-invasive ventilation failure within 72 h of treatment initiation, and no significant differences were observed between the two groups in prespecified safety outcomes. These findings suggest that, in settings where LISA is difficult to implement due to technical or economic constraints, NDT-InSurE may serve as a practical and feasible alternative across different levels of healthcare institutions, particularly in resource-limited settings. Future larger, multicenter studies are warranted to confirm its long-term efficacy and safety.

## Data Availability

The raw data supporting the conclusions of this article will be made available by the authors, upon request and without undue reservation. Requests to access the data can be directed to: Jin Gao, kaola25@126.com.
