# Supplementary material for: A novel double-lumen tracheal tube-assisted InSurE versus LISA in preterm infants with RDS: a multicenter randomized trial
Source: Front Pediatr. 2026 Aug 6;14:1869213. doi: 10.3389/fped.2026.1869213 (PMC13490172; doi:10.3389/fped.2026.1869213)
Supplement: Supplementary file 1 [file Supplementaryfile3.pdf]

Supplementary Materials

Table S1 Secondary outcomes in NDT-InSurE versus LISA groups in infants ≤28 weeks' gestation

Parameters	NDT-InSurE group (n=5)	LISA group (n=10)	Z	*P* value
Procedure duration				
Total procedure duration, min, median (P25, P75)	20.0 (15.0,23.5)	12.5 (5.0,21.25)	-.122	0.903
Intubation time, s, median (P25, P75)	7.0(5.0,11.95)	12.5 (8.5,21.75)	-1.601	0.109
Invasive ventilation duration, h, median (P25, P75)	198.0 (101.5,216.5)	78.25 (29.5,191.75)	-1.287	0.198
Non-invasive ventilation duration, h, median (P25, P75)	1152.0 (726.0,1191.0)	582.0 (180.0,1005.0)	-1.837	0.066
Oxygen therapy duration, h, median (P25, P75)	567.0 (306.0,1026.0)	412.5 (260.75,547.25)	-1.347	0.178
Total hospitalization time, d, median (P25, P75)	85.0 (47.0,86.5)	60.0 (9.75,88.25)	-1.166	0.244
Ventilation and oxygenation				
SpO_2 after PS, %, median (P25, P75)	92 (90,93)	94 (92,95)	-1.833	0.067
FiO_2 before PS %, median (P25, P75)	40.0 (29.5,62.5)	35.0 (29.25,40.00)	-0.747	0.455
FiO_2 after PS, %, median (P25, P75)	35.0 (30.0,37.5)	30.0 (26.5,33.0)	-2.075	0.038
pH before PS, median (P25, P75)	7.12 (7.08,7.24)	7.27 (7.21,7.31)	-1.719	0.086
pH after PS, median (P25, P75)	7.29 (7.26,7.32)	7.42 (7.27,7.44)	-1.143	0.253
PaO_2 before PS, mmHg, median (P25, P75)	80.0 (66.0,92.5)	85.5 (65.43,98.27)	-0.247	0.805
PaO_2 after PS, mmHg, median (P25, P75)	76.0 (59.0,86.0)	70.35 (52.50,79.00)	-1.000	0.317
$PaCO_2$ before PS, mmHg, median (P25, P75)	38.0 (31.0,56.0)	38.47 (29.25,43.25)	-0.675	0.499
$PaCO_2$ after PS, mmHg, median (P25, P75)	32.0 (27.5,36.0)	34.5 (25.5,41.5)	-0.268	0.788

Table S2 Secondary outcomes in NDT-InSurE versus LISA groups in infants >28 to 30 weeks' gestation

Parameters	NDT-InSurE group (n=27)	LISA group (n=23)	Z	*P* value
Procedure duration				
Total procedure duration, min, median (P25, P75)	30.0 (20.0,46.5)	30.0 (20.0,34.2)	-.562	0.574
Intubation time, s, median (P25, P75)	12.0 (8.0,17.0)	18.0 (11.0,20.0)	-1.513	0.130
Invasive ventilation duration, h, median (P25, P75)	233.0 (214.0,323.0)	375.0 (287.0,406.0)	-3.993	<0.001
Non-invasive ventilation duration, h, median (P25, P75)	507.0 (239.5,828.0)	323.5 (180.75,923.0)	-0.756	0.449
Oxygen therapy duration, h, median (P25, P75)	344.0 (154.5,409.5)	237.0 (168.0,306.0)	-0.679	0.497
Total hospitalization time, d, median (P25, P75)	60.0 (55.0,74.0)	60.0 (45.75,71.75)	-.098	0.922
Ventilation and oxygenation				
SpO_2 after PS, %, median (P25, P75)	94.00 (92.00,94.0)	93.00 (91.00,94.00)	-0.960	0.337
FiO_2 before PS %, median (P25, P75)	35.00 (30.00,80.00)	31.00 (30.00,40.00)	-1.867	0.062
FiO_2 after PS, % median (P25, P75)	30.00 (27.00,35.00)	32.00 (28.50,38.75)	-0.296	0.767
pH before PS, median (P25, P75)	7.30 (7.24,7.35)	7.32 (7.25,7.35)	-0.234	0.815
pH after PS, median (P25, P75)	7.30 (7.26,7.36)	7.36 (7.32,7.41)	-2.789	0.005
PaO_2 before PS, mmHg, median (P25, P75)	39.00 (32.00,44.00)	35.52 (32.0046.00,)	-0.652	0.514
PaO_2 after PS, mmHg, median (P25, P75)	30.00 (25.10,31.00)	32.51 (20.39.0061,)	-0.048	0.962
$PaCO_2$ before PS, mmHg, median (P25, P75)	63.00 (47.00,78.00)	69.71 (56.00,88.00)	-0.935	0.350
$PaCO_2$ after PS, mmHg, median (P25, P75)	75.00 (60.9,487.48)	86.12 (67.18,96.48)	-1.655	0.098

Table S3 Secondary outcomes in NDT-InSurE versus LISA groups in infants >30 to 32 weeks' gestation

Parameters	NDT-InSurE group (n=37)	LISA group (n=36)	Z	*P* value
Procedure duration				
Total procedure duration, min, median (P25, P75)	20.0 (15.25, 30.0)	15.0 (7.5, 26.5)	-.462	0.644
Intubation time, s, median (P25, P75)	8.0 (5.0, 12.0)	12.0 (9.0, 18.5)	-1.824	0.068
Invasive ventilation duration, h, median (P25, P75)	238.0 (166.0,302.4)	223.0 (96.0,240.0)	-1.942	0.052
Non-invasive ventilation duration, h, median (P25, P75)	168.0 (113.25,334.75)	228.0 (124.0,570.0)	-1.582	0.114
Oxygen therapy duration, h, median (P25, P75)	582.0 (128.0,654.0)	683.0 (176.0,758.0)	-3.151	0.002
Total hospitalization time, d, median (P25, P75)	46.5 (39.25,60.0)	53.0 (45.0,63.0)	-.623	0.533
Ventilation and oxygenation				
SpO_2 after PS, %, median (P25, P75)	94.00 (92.00,95.00)	93.00 (91.00,94.00)	-0.138	0.890
FiO_2 before PS %, median (P25, P75)	40.00 (30.00,40.00)	35.00 (30.00,40.00)	-0.770	0.441
FiO_2 after PS, % median (P25, P75)	30.00 (27.50,35.00)	29.00 (28.75,35.00)	-0.208	0.836
pH before PS, median (P25, P75)	7.25 (7.21,7.30)	7.28 (7.24,7.36)	-0.782	0.4340
pH after PS, median (P25, P75)	7.33 (7.28,7.34)	7.36 (7.31,7.40)	-2.704	0.007
PaO_2 before PS, mmHg, median (P25, P75)	40.00 (37.32,52.48)	38.55 (31.57,45.87)	-1.115	0.265
PaO_2 after PS, mmHg, median (P25, P75)	35.15 (28.12,38.50)	30.00 (24.76,36.47)	-1.777	0.076
$PaCO_2$ before PS, mmHg, median (P25, P75)	88.90 (73.56,96.53)	83.98 (71.07,95.00)	-1.683	0.092
$PaCO_2$ after PS, mmHg, median (P25, P75)	71.82 (57.90,83.10)	85.11 (64.36,94.31)	-0.308	0.758
