# Supplementary material for: A novel double-lumen tracheal tube-assisted InSurE versus LISA in preterm infants with RDS: a multicenter randomized trial
Source: Front Pediatr. 2026 Aug 6;14:1869213. doi: 10.3389/fped.2026.1869213 (PMC13490172; doi:10.3389/fped.2026.1869213)
Supplement: Supplementary file 2 [file Supplementaryfile1.docx]

**Supplementary material 1**

Case Report Form

NISA Multi-Center Study

Enrollment number:

Enrollment group: New tube group□	LISA group□

Enrollment center:

□ Kunming Children's Hospital

□ Qujing Maternal and Child Health-care Hospital□ Zhaotong People's Hospital

□ Wenshan People's Hospital

□ Yuxi Children's Hospital

□ Baoshan People's Hospital

**Please read the following instructions carefully before completing the form.**

Instructions for completing the case report form

1. Screening qualified patients to complete the case report form.

2. Case report form must be filled out accurately, clearly, not arbitrarily altered, the error is corrected with a horizontal line drawn in the center, for example: ~~99.6~~, and then fill in the correct figures and text next to it, and sign the name of the modification of the initials and the time of modification (do not use an eraser, correction fluid to cover up or draw many lines).

3. Fill out the record always with a water-based pen or carbon signature pen.

4. For all items with “□” in the table, please mark “O” on the conforming entries. All columns in the table should be filled in with the corresponding words or figures, and should not be left blank.

5. All items are not checked, omitted or cannot be completed by the organization for any reason, please fill in “NK (unknown)”; if the item is not needed or not appropriate, fill in “NA (not applicable)”.

6. If an adverse event occurs, record the time of occurrence, severity and duration of the adverse event, measures taken and regression. Please report to the person in charge of the clinical study at the research unit in time.

**NISA Multi-Center Study ---- Clinical Trial Flowchart**

| Program | Screening Period | | Before PS | Complete PS injection for 6 hours | Discharge | Corrected 12 months age completed follow up |
| --- | --- | --- | --- | --- | --- | --- |
|  | Preterm infants | Day of admission |  |  |  |  |
| Whether met inclusion criteria | √ |  |  |  |  |  |
| Signed informed consent | √ |  |  |  |  |  |
| Randomized grouping of reporting centers |  | √ |  |  |  |  |
| Demographic information |  | √ |  |  |  |  |
| Family information |  | √ |  |  |  |  |
| Mother's maternal history |  | √ |  |  |  |  |
| Neonatal situation |  | √ |  |  |  |  |
| Admission Condition |  | √ |  |  |  |  |
| PS Using |  |  | √ |  |  |  |
| After PS use |  |  |  | √ |  |  |
| Situation during hospitalization |  |  |  |  | √ |  |
| PMA 12 months |  |  |  |  |  | √ |

**Based on the history and physical examination, please confirm the following**

| Inclusion criteria | Yes | No |
| --- | --- | --- |
| (1) Those whose gestational age at birth is less than 32 weeks (counting from the first day of the last menstrual period) and whose pregnancy is terminated before the 32nd week of gestation (inclusive), regardless of sex. |  |  |
| (2) Transferred to the NICU of the study unit within 6 hours of birth and given noninvasive respiratory support with FiO2 > 30% to maintain saturation between 90-95% |  |  |
| (3) Respiratory support mode:CPAP、SNIPPV、BiPAP |  |  |
| (4) Diagnosed with NRDS based on clinical presentation and chest imaging features |  |  |
| (5) Written informed parental consent was obtained |  |  |
| If the answer to any of the above is “No”, the subject cannot participate in the study. | | |

| Exclusion criteria | Yes | No |
| --- | --- | --- |
| (1) Severe congenital anomalies, known complex congenital heart disease, or congenital anomalies of lung development |  |  |
| (2) Chromosomal abnormalities or inherited metabolic disorders |  |  |
| (3) Neuromuscular disorders affecting respiratory function |  |  |
| (4) Grade 3 or 4 intraventricular hemorrhage (IVH) |  |  |
| (5) Receiving mechanical ventilation in the delivery room or requiring immediately mechanical ventilation support by clinical judgment |  |  |
| (6) Exogenous surfactants have been applied prior to enrollment |  |  |
| (7) Transferred out of the NICU before randomization |  |  |
| (8) Requires surgical treatment |  |  |

1. **General information**

| 1.1 Patient name |  |
| --- | --- |
| 1.2 Hospitalization number |  |
| 1.3 Contact information (phone or WeChat) |  |

**2、Basic information on mother**

| 2.1（1）Parents' age | Father years Mother years |
| --- | --- |
| （2）Mother's births |  |
| （3）Adverse maternal history | Spontaneous Miscarriage□Fetal Miscarriage□Maternal Miscarriage□Gravida□Stillbirth pregnancy□Stillbirth□Prenatal Hemorrhage□Other□ |
| 2.2 Mother's literacy level | Elementary school□Junior high school□High school□University□Master's degree and above□Illiterate□ |
| 2.3 Mother's profession | Student□Commercial□Worker□Manager□Official□Employee□Professional□Individual □Military□Unemployed□Farmer □Other |
| 2.4 Mother's weight before the current pregnancy | kg |
| 2.5 Mother's weight before the current delivery | kg |
| 2.6 Mothers' pre-pregnancy underlying diseases |  |
| 2.7 Father's literacy level | Elementary school□Junior high school□High school□University□Master's degree and above□Illiterate□ |
| 2.8 Father's profession | Student□Commercial□Worker□Manager□Official□Employee□Professional□Individual □Military□Unemployed□Farmer □Other |

**3、Basic information**

| 3.1premature rupture of membranes | No□ Yes□ hr |
| --- | --- |
| 3.2 Gestational diabetes |  |
| （1）Gestational age at diagnosis | weeks |
| （2）Maximum blood glucose level | mmol/L |
| （3）Gestational diabetes treatment |  |
| （4）Prenatal hospitalization of newborns |  |
| 3.3 Prenatal glucocorticoid use |  |
| （1）Course of glucocorticoid use (days) | Yes□(Session completed within 1 week before delivery□Session completed more than 1 week before delivery□Session completed before delivery, date unknown□Partial session completed 24 hours before delivery□Session and date unknown□)  No□ Unkonwn□ |
| （2）Time from glucocorticoid administration to birth | hours |
| 3.4 Placenta praevia | No□ Yes□ |
| （1）Placenta praevia with placental implantation | No□ Yes□ |
| （2）Duration of prenatal hospitalization before birth of the newborn | No□ Yes□ |
| 3.5 Hypertensive disorders of pregnancy | No□ Yes□ |
| （1）Highest blood pressure: systolic blood pressure | mmHg |
| （2）Diastolic blood pressure | mmHg |
| （3）Prenatal hospitalization of newborns | No□ Yes□ |
| 3.6 Intrahepatic cholestasis in pregnancy | No□ Yes□ |
| （1）Bile acid level | mg/L |
| （2）ALT | U/L |
| （3）AST | U/L |
| （4）Prenatal hospitalization of newborns | No□ Yes□ |
| 3.7 Test-tube baby | No□ Yes□ |
| 3.8 Mother's drug addiction | No□ Yes□ |
| 3.9 Smoking | No□ Yes□ |
| 3.9.1 Drinking | No□ Yes□ |
| 3.10 Oral folic acid during pregnancy | No□ Yes□ |
| 3.11 Prenatal examination |  |
| （1）Regulation of prenatal examinations | No□ Yes□ |
| （2）Maternity hospitals and grades | Level II hospitals□ Level III hospitals□ |
| 3.12 Severe vomiting during pregnancy | No□ Yes□ |
| 3.13 Other risk factors during pregnancy |  |
| （1）Systemic lupus erythematosus in pregnancy | No□ Yes□ |
| （2）Hypothyroidism in pregnancy | No□ Yes□ |
| （3）Hyperthyroidism in pregnancy | No□ Yes□ |
| （4）Mental illness in pregnancy | No□ Yes□ |
| （5）Pregnancy combined with other diseases | No□ Yes□ |
| 3.14 Milk choking | No□ Yes□ |

4. **Information about the child**

| 4.1（1）Gestation week | week day |
| --- | --- |
| （2）Birth weight | g |
| （3）Whether twin/multiple fetuses | Yes□,number of fetuses No□ |
| 4.2 Date of birth | year month day hour minute |
| 4.3 Sex | man□ female□ |
| 4.4 Mode of delivery | Normal labor□ Cesarean section□ Forceps□ Breech delivery□ |
| 4.5 Apgar score：1min-5min-10min |  |
| 4.6 Admission temperature（℃） |  |
| 4.7 Heart rate (beats per minute) |  |
| 4.8 Respiration (breaths/min) |  |
| 4.9 SpO2（%） |  |
| 4.10 Weight（g） |  |
| 4.11 Height（cm） |  |
| 4.12 Head circumference（cm） |  |
| 4.13 Blood pressure（mmHg） |  |
| 4.14 Possible causes of preterm birth | Fetus: Clouding of amniotic fluid□ Premature rupture of membranes□ Intrauterine distress□ Abnormal fetal heartbeat□ Abnormal fetal position□ Twin fetuses□ Multiple fetuses□ Hydramnios□ Oligohydramnios□  Placenta, Uterus and Umbilical Cord: Placenta previa□ Placental abruption□ Umbilical cord causes□ Uterine causes□ Cervical causes  Maternal (pregnancy complications and comorbidities) Hypertension (gestational hypertension□pre-eclampsia□chronic hypertension combined with pre-eclampsia□chronic hypertension combined in pregnancy□) Diabetes □[Pregestational diabetes mellitus (PGDM)□Gestational diabetes □Thyroid disease□Nephropathy□Hepatopathy□Reproductive tract infections□Systemic infections□Anemia□Surgery□Overexertion□Increased mood swings□Unintentional injuries□Habitual miscarriage□Other cause of preterm birth□Unknown□ |

5. **Diagnosis**

| 5.1 Primary Diagnosis: |  |
| --- | --- |
| 5.2 Secondary Diagnosis:（1） | ① |
| （2） | ② |
| （3） | ③ |

6. **Number of intubations**

| 6.1The first time: (1) Model (inner diameter): | mm |
| --- | --- |
| （2）Intubation time | s |
| （3）The depth of the oral tract | cm |
| （4）Title of intubation physician | Resident □ Attending □ Senior □ |
| （5）Time engaged in the profession | Years |
| 6.1.1 Whether the drug is dripped into the trachea | No□ Yes□ |
| 6.1.2 Sources of drug dripped | Domestic□ Imported□ |
| 6.1.3 Whether drug reflux occurs | No□ Yes□ |
| 6.1.4 Whether there is bleeding from airway injury | No□ Yes□ |
| 6.1.5 Respiratory depression while intubation | No□ Yes□ b/minute |
| 6.1.6 Bradycardia while intubation | No□ Yes□ b/minute |
| 6.1.7 Tachycardia while intubation | No□ Yes□ b/minute |
| 6.1.8 Laryngospasm while intubation | No□ Yes□ |
| 6.1.9 Coughing and wheezing after extubation | No□ Yes□ |
| 6.1.10 Oral mucous membrane damage | No□ Yes□ |
| 6.1.11Repeated choking after extubation | No□ Yes□ b/hr |
| 6.1.12Whether or not a second intubation was performed, if so please complete the items in the preamble to fill in |  |
| 6.2 The second time: (1) Model (inner diameter): | mm |
| （2）Intubation time | s |
| （3）The depth of the oral tract | cm |
| （4）Title of intubation physician | Resident □ Attending □ Senior □ |
| （5）Time engaged in the profession | Years |
| 6.1.1 Whether the drug is dripped into the trachea | No□ Yes□ |
| 6.1.2 Sources of drug dripped | Domestic□ Imported□ |
| 6.1.3 Whether drug reflux occurs | No□ Yes□ |
| 6.1.4 Whether there is bleeding from airway injury | No□ Yes□ |
| 6.1.5 Respiratory depression while intubation | No□ Yes□ b/minute |
| 6.1.6 Bradycardia while intubation | No□ Yes□ b/minute |
| 6.1.7 Tachycardia while intubation | No□ Yes□ b/minute |
| 6.1.8 Laryngospasm while intubation | No□ Yes□ |
| 6.1.9 Coughing and wheezing after extubation | No□ Yes□ |
| 6.1.10 Oral mucous membrane damage | No□ Yes□ |
| 6.1.11 Repeated choking after extubation | No□ Yes□ b/hr |
| 6.2.13 Third intubation |  |

7. **Drug administration**

| 7.1 Age of first intratracheal surfactant administration | hours minutes |
| --- | --- |
| 7.2 Total operating time: from the time the laryngoscope is placed in the mouth to the time the catheter is withdrawn at the end of drug injection | minutes |
| 7.3 Number of intubations attempted before successful administration: when the laryngoscope was delivered into the neonate's mouth, an attempt was considered to have been generated whether or not a catheter was inserted, and the attempt was judged to be over when both the laryngoscope and catheter were removed from the mouth | times |
| 7.4During drug administration (1) Bradycardia (heart rate <100 beats/min) | No□ Yes□ b/minute |
| （2）Tachycardia (heart rate >200 beats/min) | No□ Yes□ b/minute |
| （3）Low saturation (SpO2 ≤ 80%) | No□ Yes□ b/minute |
| 7.5.1 The gastric tube was drawn back after the surfactant had completed administration and observed for the presence of PS-like material; | No□ Yes□ |
| 7.5.2 Chest radiographs before and 6 hours after surfactant administration were compared, and cases with inconsistent improvement in bilateral lung lesions were judged to have been unevenly administered | No□ Yes□ |
| 7.6 Surfactants before administration（1）SpO2（%） |  |
| （2）FiO2 | % |
| （3）pH |  |
| （4）PaCO2（mmHg） |  |
| （5）PO2（mmHg） |  |
| 7.7Surfactants after administration（1）SpO2（%） |  |
| （2）FiO2 | % |
| （3）pH |  |
| （4）PaCO2（mmHg） |  |
| （5）PO2（mmHg） |  |

8. **Ventilation**

| 8.1 Non-invasive ventilation mode before PS | CPAP□ NIPPV□ BIPAP□ |
| --- | --- |
| 8.1.1 Age at first non-invasive ventilation | hours minutes |
| 8.3.1 Mode of non-invasive ventilation after PS (if there is a change within 72 hours please fill in the new mode of ventilation here) |  |
| （1）PIP(CPAP mode filled NA)(cmH2O) |  |
| （2）Ti(s) |  |
| （3）PEEP（cmH2O） |  |
| （4）FiO2 | % |
| 8.3 Ventilation mode switching |  |
| 8.3.1 Initial Mode-Convert First Mode-Convert Second Mode ..... (fill in the duration (hours) until atmospheric oxygen supply) |  |
| 8.3.2 Mechanical ventilation: mode (during hospitalization) |  |
| 8.3.3(1) Highest PIP/MAP (cmH2O) |  |
| （2）Highest oxygen concentration | % |
| （3）Ventilator up time | hours |
| （4）Post-mechanical ventilation respiratory support modalities |  |
| （5）Respiratory support duration | hours |
| （6）Oxygen using time | hours |
| 8.4 Non-invasive post-oxygenation mode |  |
| 8.4.1Duration of non-invasive ventilation | hours |
| 8.5 Transnasal high flow: (1) Flow rate | L/min |
| （2）oxygen concentration FiO2 | % |
| （3）Switch to box/nasal tube oxygen after ___ days | days |
| （4）Oxygen withdrawal on ___ days | days |
| 8.6 Oxygen in tank: (1) Flow rate | L/min |
| （2）oxygen concentration FiO2 | % |
| （3）Oxygen withdrawal on ___ days | days |
| 8.7 Nasal tube oxygen: (1) Flow rate | L/min |
| （2）oxygen concentration FiO2 | % |
| （3）Oxygen withdrawal on ___ days | days |

9. **Complications**

| 9.1Intracranial hemorrhages | No□ Yes□ |
| --- | --- |
| 9.1.2 Intracranial hemorrhages grade | I□ II □III□ IV□ |
| 9.1.3 Time of occurrence (corrected gestational age w) |  |
| 9.2 NEC | No□ Yes□ |
| 9.2.1 NEC grade | Bell I□ IIa □IIb □III□ |
| 9.2.2 Time of occurrence |  |
| 9.3 Air leakage in the lungs | No□ Yes□ |
| 9.3.1 Air leakage state | Unilateral□Bilateral□Mediastinal pneumoperitoneum□Other□ |
| 9.3.2 Time of occurrence |  |
| 9.4 Retinopathy of prematurity (ROP) | No□ Yes□ |
| 9.4.1 Results of fundus screening |  |
| 9.4.2 Time of occurrence |  |
| 9.5 PVL | No□ Yes□ |
| 9.5.1Time of PVL occurrence |  |
| 9.6 Pulmonary hemorrhage | No□ Yes□ |
| 9.6.1 Time of occurrence |  |
| 9.7 BPD | No□ Yes□ |
| 9.7.1 BPD grade | Mild□Moderate□Severe□ |
| 9.7.2 Time of occurrence |  |
| 9.8 Death |  |

10. **Secondary infections**

| 10.1 Secondary infections | No□ Yes□ |
| --- | --- |
| 10.2 Location of secondary infection |  |
| 10.3 Infectious agent |  |
| 10.4 Time of occurrence |  |

11. **Discharge**

| 11.1 Mode of discharge | Medically discharged□ Signed discharged with improvement□ Signed discharged with no improvement cannot survivable□ |
| --- | --- |
| 11.2 Duration of hospitalization | days |
| 11.3 Discharge weight | kg |
| 11.4 Discharged with oxygen | No□ Yes□ |

12. **Follow-up**

| 12.1 Oxygen discharge with oxygen withdrawal time | days |
| --- | --- |
| 12.2 Follow-up 1 month after discharge:(1) Whether repeated dyspnea | No□ Yes□ |
| （2）Whether readmitted within 1 month | No□ Yes□ times |
| 13.2 Respiratory outcomes were observed over 1 year of follow-up: (1) number of upper respiratory tract infections in the child during the first 12 months of life (corrected age) | times |
| （2）Hospitalized for respiratory problems | No□ Yes□ times |
| （3）Wheezing |  |
| （4）Weight and growth at follow-up | kg cm |
| （5）Death | No□ Yes□ |

13. **Research summary**

Patient's date of admission: Year	Month	Day

Date of discharge：	Year	Month	Day

End of study: Year	Month	日(The last follow-up time)

Adverse events occur? No□ Yes□→Resolved□ Unsolved□→Monitor adverse events until stabilization or resolution

Did the patient complete the clinical study according to the protocol plan? Yes □ No□→Please complete the following items

The date the patient ends the study: Year	Month	Day

The main reasons for ending the study was (choose one)

Guardian's request to withdraw from the trial……………………… □

Poor compliance…………………………………… □

Loss of follow-up………………………………………… □→Reason：

Research end…………………………………… □

Other………………………………………… □→Reason：
