# Supplementary material for: A novel double-lumen tracheal tube-assisted InSurE versus LISA in preterm infants with RDS: a multicenter randomized trial
Source: Front Pediatr. 2026 Aug 6;14:1869213. doi: 10.3389/fped.2026.1869213 (PMC13490172; doi:10.3389/fped.2026.1869213)
Supplement: Supplementary file 3 [file Supplementaryfile2.docx]

**Supplementary material 2**

Informed consent

Program title: Novel dual-lumen endotracheal tube in preterm infants with respiratory distress syndrome: protocol for a randomized controlled trial (NISA)

Informed consent version number: November 8, 2023

Institution(s): Kunming Children's Hospital, Qujing Maternal and Child Health-care Hospital, Zhaotong People's Hospital, Wenshan People's Hospital, Baoshan People's Hospital, Yuxi Children's Hospital, Yunnan, China

Principal Investigator(s): Jin Gao, Chuanfeng Li, Hanmei Xiong, Weihong Yu, Panrong Nie, Jie Yang

Patient's Name:

Patient's initials (pinyin initials capitalized):

Patient's address:

Patient's hospitalization number:

Patient phone number:

Dear	：

We would like to invite your child to participate as a subject in a clinical trial. This informed consent form provides you with information to help you decide whether to participate in this clinical trial. Please take some time to read the following carefully and discuss any unclear questions or terms with the physician. Contact Doctor: Jane Gao

Phone number: 0871-63309356

Your participation in this study is completely voluntary. This study has been reviewed by the Ethics Committee.

1. **What was the purpose of the study?**

A randomized controlled trial to compare the efficacy of a novel dual-lumen endotracheal intubation-surfactant-extubation (InSurE) with minimally invasive surfactant administration (LISA) in the treatment of respiratory distress syndrome in preterm infants.

2. **How the study conducted?**

(1) Case data collection will be completed from parents of all patients entering the study, including family address and family status.

(2) Children with composite enrollment criteria will be randomly assigned to 2 groups and given pulmonary surfactant treatment with different catheters.

(3) The children will be followed up for 1 year after discharge to observe clinical outcomes.

3. **What was the exact process of the study?**

(1) Case data collection will be completed from parents of all patients entering the study, including family address and family status.

(2) Children with composite enrollment criteria will be randomly assigned to 2 groups and given pulmonary surfactant treatment with different catheters.

(3) The children will be followed up for 1 year after discharge to observe clinical outcomes.

4. **What did a subject need to do in the study?**

The subjects of our study were neonates, and parents were asked to provide truthful information about the physical condition of the mother and the child.

5. **What are the benefits of participating in the study?**

Child and society will likely benefit from this study. We are comparing the differences between the 2 catheters (one of which has received worldwide approval), and if there is no significant difference between the new catheter and the LISA catheter, then clinicians will have an additional option for the use of lung surfactant.

6. **What are the risks of participating in the study?**

Our study subjects are neonates and the treatment protocols follow the appropriate clinical protocols without any risks or adverse effects.

7. **Will participation in the study be remunerated? (If so, how)**

You will not receive any payment for participating in this trial or this study.

8. **Is there any fee for participation in the study?**

There is no any cost to neonates and parents who participate in the studies included in this study.

9. **How to deal with injuries received while participating in the study?**

Our study does not use a new method, only a new catheter, so no harm can occur. Even though you have signed this informed consent form, you can retain all of your legal rights and interests.

10. **Does subject have to participate in the study?**

Before the experiment, please make a detailed understanding of the clinical trial, the hospital and physicians are obliged to provide you with information related to the clinical trial, to explain your concerns, and then you voluntarily decide whether to participate in the clinical trial treatment. You have the right to withdraw from any stage of the study, and withdrawing from the study will not affect the regular treatment of you.

11. **Responsibility for confidentiality**

Any results and information obtained from this study will belong to the implementer of the clinical validation program and the medical institution and will be used without compensation, but your legitimate rights and interests will not be infringed upon by this study. Your personal data will be kept confidential by our hospital, and the Ethics Committee and the implementer of the study can access your data, but neither of them will disclose its content to the public, and your identity will not be disclosed unless required by law. The results of the study will be published for scientific purposes without revealing your identity.

12. **Modalities for project complaints**

When there are questions about the information and rights of study participants, as well as damages related to the study, participants may contact the Clinical Trial Ethics Committee of Kunming Children's Hospital at 63309095.

13. **Informed Consent Signature - Consent Signature Sheet**

If you fully understand the contents of this research project and agree to participate in this study, you will sign this informed consent form in duplicate, with one copy to be retained by the investigator and one copy to be retained by the subject or the delegate.

Clinical Research Project Title: Preterm Infant Respiratory Distress Syndrome Using Novel Dual Lumen Endotracheal Intubation-Surfactant-Extubation (InSurE) and Minimally Invasive Surfactant Administration (LISA): A Randomized Controlled Study.

Subject Consent Statement:

1. I confirm that I have read and understood the informed consent form for this study. Any problems that may arise during the course of the study and how they will be solved have been explained to me, and I have been given the opportunity to ask my questions.

2、 I have been informed that participation in the study is voluntary and that refusal to participate will not jeopardize any of the benefits to which I am entitled.

3. I have been informed that the physicians participating in this study, the person in charge of this work at Kunming Children's Hospital, and the Medical Ethics Committee of Kunming Children's Hospital have the right to review the study records and case data. I agree the abovementioned persons may have direct access to my study records, and I understand the abovementioned information will be treated in a confidential manner.

4. I agree to participate in this study

The subject's name in standard script:	Signature:

Date: Contact number:

**(Signature should be the guardian when the subject is incapable of civil behavior; signature should be the subject and his/her guardian when he/she does not have limited civil capacity; signature should be the notary when the subject or his/her guardian lacks the ability to read)**

Guardian's name in standard script:	Signature:

Date: Contact number:

Notary’s name in standard script:	Signature:

Date: Contact number:

The following is completed by the physician performing the informed consent process.

Researcher's statement: I confirm that the nature, purpose, requirements, and possible risks of this study have been explained and discussed with the patient, and other available treatment options have been explored at the same time. I have ensured that a copy of this Subject Informed Consent Form has been given to the subject.

Researcher's name in standard script:	Signature:

Date: Contact number:
